# A Survey of Personal Health Habits, Wellness, and Burnout in Practicing Orthopaedic Surgeons—Are We Taking Care of Ourselves?

**DOI:** 10.5435/JAAOSGlobal-D-22-00099

**Published:** 2023-05-09

**Authors:** Jeremy C. Thompson, Michael J. VanWagner, Aaron C. Spaulding, Benjamin K. Wilke, Bradley S. Schoch, Luke S. Spencer-Gardner, Cameron K. Ledford

**Affiliations:** From the Department of Orthopedic Surgery, Mayo Clinic, Jacksonville, FL.

## Abstract

The personality traits of those who become orthopaedic surgeons may also lead to overwork, work-life balance issues, and burnout. Health and wellness practices of orthopaedic surgeons have not been widely explored. This study evaluated the personal health habits, wellness, and burnout of practicing orthopaedic surgeons in the United States.

**Methods:**

An anonymous self-assessment survey was completed by 234 practicing orthopaedic surgeon alumni from two large residency programs. The survey assessed exercise habits according to Centers for Disease Control and Prevention recommendations, compliance with preventive medical care practices according to the United States Preventive Services Task Force, prioritization of occupational wellness strategies, and the presence of burnout via an adapted Maslach Burnout Inventory. Survey responders' mean age was 52 years, 88% were male, and 93% had a body mass index <30 kg/m^2^. Surgeons were stratified according to practice type, years in practice, and subspecialty.

**Results:**

Among orthopaedic surgeons, compliance with aerobic and strength exercise recommendations was 31%. Surgeons in academic practice were significantly (*P* = 0.007) less compliant with exercise recommendations (18%) compared with private (34%) or employed (43%) practicing surgeons. Most (71%) had seen their primary care provider within 2 years and were up to date on age-appropriate health care screening including a cholesterol check within 5 years (79%), colonoscopy (89%), and mammogram (92%). Protecting time away from work for family/friends and finding meaning in work were the most important wellness strategies. The overall burnout rate was 15% and remained not significantly different (*P* > 0.3) regardless of years in practice, practice type, or subspecialty.

**Conclusion:**

This survey study identifies practicing orthopaedic surgeons' health habits and wellness strategies, including limited compliance with aerobic and strength exercise recommendations. Orthopaedic surgeons should be aware of areas of diminished personal wellness to improve quality of life and avoid burnout.

Burnout is defined as a clinical syndrome of emotional exhaustion, depersonalization, and a low sense of personal accomplishment.^1^ These feelings can be expressed by lost enthusiasm for one's work, cynicism, physical and mental exhaustion, poor judgment, guilt, and feelings of ineffectiveness.^2^ Physician burnout has serious implications as it has been associated with medical errors, unprofessional conduct, reduced quality of patient care as well as alcohol abuse, poor physical quality of life, and reduced career satisfaction.^3,4,5,6,7^

The orthopaedic surgery subspecialty has been shown to be at higher risk for burnout, with rates reported as high as 57% in US orthopaedic residents, fellows, and attendings.^8,9,10,11,12,13^ The orthopaedic surgery specialty attracts highly motivated, dedicated individuals who often work long hours and make personal wellness sacrifices for patient care. Although these characteristics and behaviors may contribute to professional success, they can also promote an unhealthy lifestyle predisposing to burnout.^14-16^

Attention to personal health habits has been recognized as protective against burnout, yet very little is known about orthopaedic surgeons' well-being and health practices. This survey study aimed to evaluate the personal health habits, wellness, and burnout of practicing orthopaedic surgeons in the United States.

## Methods

After institutional review board approval, an anonymous self-assessment survey was electronically mailed to all registered orthopaedic surgery residency and fellowship alumni from two large academic training programs. Surveys were sent once a week with a repetitive reminder for 3 weeks and responses stored in a secured internal database. Participation was elective, and results remained anonymous. Responses from nonpracticing, retired, or nonsurgical clinical practice were excluded. A total of 792 orthopaedic surgeons were sent the survey, 355 (45%) opened the survey, and 234 (30%) fully completed the survey used for the analysis.

The survey included 37 questions assessing demographic information, practice characteristics, personal health habits, routine medical care and health screening practice, wellness strategies, and quality of life measurements. Practice characteristics were reported as practice type (private, academic, and hospital employed), number of years in practice, and primary subspecialty (Table 1). The orthopaedic surgeon survey responders' mean age was 52 years (SD + 12 years), 88% were male, and 55% reported a normal body mass index (18.5 to 25 kg/m^2^). A wide variation of practice type and years in practice was seen, including private practice (50%) and practice for >20 years (48%) being most common. All orthopaedic subspecialties were represented with the highest survey distribution represented by hip and knee reconstruction (30%), hand (18%), and sports medicine (14%) surgeons.

**Table 1:** Demographics of Practicing Orthopaedic Surgeon Survey Responders

Age (mean yrs, SD)	52 (SD + 12)
BMI (kg/m^2^), n (%)	
Underweight (<18.5)	0 (0)
Normal (18.5 to <25)	128 (55)
Overweight (25 to <30)	90 (38)
Obese (>30 to <40)	14 (6)
Severely obese (>40)	0 (0)
Missing	2 (1)
Sex, n (%)	
Male	205 (88)
Female	27 (12)
Missing	2 (1)
Years in practice, n (%)	
<5 yr	41 (18)
5-10 yr	29 (12)
10-20 yr	50 (22)
>20 yr	113 (48)
Missing	1 (<1)
Practice type, n (%)	
Private practice	117 (50)
Academic or university	79 (34)
Hospital employed	37 (16)
Missing	1 (<1)
Subspecialty, n (%)	
Spine	13 (6)
Pediatrics	11 (5)
Trauma	9 (4)
Oncology	4 (2)
Sports medicine	33 (14)
Hand	41 (18)
Shoulder/elbow	14 (6)
Hip and knee reconstruction	71 (30)
Foot and ankle	17 (8)
General	21 (9)

BMI = body mass index

Personal health habit questions sought information about tobacco use, caffeine intake, and aerobic and/or strengthening exercise participation. The intensity and duration of exercise were compared according to the Centers for Disease Control and Prevention (CDC) recommendations of >150 minutes per week of moderate-intensity or >75 minutes of vigorous aerobic activity and at least 2 days per week of muscle-strengthening activity.^17^

Routine medical care was assessed by requesting information regarding the most recent participation in primary care and dentist visits and applicable preventive screening tests for cholesterol, colorectal cancer, and breast cancer. For routine primary care visits, opinions vary, but it can generally be accepted that healthy adults should obtain this evaluation every 1 to 3 years. Thus, surgeons were considered noncompliant if they had not seen a primary care provider in greater than 3 years.^18,19^ For dentistry, the American Dental Association recommends 6 months but a minimum 1-year interval follow-up to prevent and treat oral disease.^20-22^ Respondents were deemed compliant (<1 year), partially compliant (1 to 2 years), or non-compliant (>2 years) in respect to their dental health. The CDC recommends cholesterol screening to be done at least every 5 years, and adherence to this interval evaluation was evaluated.^23^ For preventive screening, survey responders were assessed on their compliance according to the United States Preventive Services Task Force recommendations were used for colorectal and breast cancer screening—all if age appropriate and varied according to risk factors and recommendation level^24,25^ (Table 2).

**Table 2:** Summary of Recommendations for Screening

Topic	Organization	Recommendation	Grade
Primary care screening visit	N/A	Health maintenance examination every 1-3 yr in healthy adults	None
Dental screening visit	ADA	Dental cleaning and screening examination every 6 mo but a minimum 1-yr interval follow-up for the prevention and treatment of oral disease	None
Cholesterol screening	CDC	Every 5 yr for people aged 20 yr or older who are at low risk for cardiovascular disease^a^	None
Breast cancer screening	USPSTF	Biennial screening mammography for women aged 50-74 yr	B^b^
		Biennial screening mammography for women aged 40-49 yr	C^b^
Colorectal cancer screening^c^	USPSTF	Screening for persons aged 50-75 yr	A^b^
		Screening for persons aged 45-49 yr	B^b^

ADA = American Dental Association, CDC = Centers for Disease Control and Prevention, USPSTF = United States Preventive Services Task Force

a Cholesterol screening should be done more frequently than every 5 years for people with cardiovascular disease risk factors.

b USPSTF recommendations: A = recommends service (high certainty that net benefit is substantial); B = recommends service (high certainty that net benefit is moderate and/or moderate certainty that net benefit is moderate to substantial); C = recommends selectively offering or providing service to an individual based on professional judgment and/or patient preference (at least moderate certainty that the net benefit is small).

c Colonoscopy every 10 years or CT colonography every 5 years or flexible sigmoidoscopy every 5 years.

Orthopaedic surgeons were also asked to rate a variety of wellness promotion strategies such as personal philosophies, self-care, relationships, and work attitudes. The importance of each strategy could be selected as not important, minimally important, moderately important, or essential, and each response was given a numeric score to allow a rank across the survey responders. The adapted Maslach Burnout Inventory (MBI) was used to assess for symptoms of physician burnout.^5,26-28^ The tool consists of two single-item measures: (1) emotional exhaustion assessing how often surgeons feel burned out from their work and (2) depersonalization was assessing feelings of callousness toward other people in their current work environment. Burnout was defined when survey responders demonstrated both emotional exhaustion and depersonalization.

Descriptive statistics were used to characterize population demographics. Study demographics and outcomes were summarized using frequency and percentages for categorical variables, and continuous variables were summarized by mean and SDs. Subgroup analyses were conducted to identify differences between healthy habits and respondents: (1) practice type; (2) the number of years in practice; and (3) subspecialty. Differences between groups were assessed by Pearson chi-square, Kruskal-Wallis, and Fisher exact tests as appropriate for continuous and categorical variables. All analysis was conducted using Stata 17 MP (College Station, TX).

## Results

### Personal Health Habits

The routine use of tobacco was reported to be minimal to none in 95% of orthopaedic surgeon survey responders, whereas 80% use daily caffeine. For aerobic activity, surgeons participated in limited moderate (29%) and/or vigorous (32%) exercise per week. Most (76%) reported weekly muscle-strengthening activity; however, only 31% performed >2 days. Overall, 31% of orthopaedic surgeons were compliant with both CDC aerobic and strengthening recommendations. Surgeons in academic practice were significantly (*P* = 0.007) less compliant with both CDC aerobic and strength exercise recommendations (18%) compared with private (34%) or employed (43%) practicing surgeons (Table 3). No significant difference was seen in exercise compliance when comparing the number of years in practice (*P* = 0.605) (Table 4) or subspecialty (*P* = 0.747) (Table 5).

**Table 3:** Health Habits, Routine Medical Care, and Burnout of Practicing Orthopaedic Surgeon According to Type of Practice

Health and Wellness Practice	Private Practice (n = 116)	Academic (n = 79)	Hospital (n = 37)	*P* Value
Compliant with CDC aerobic exercise recommendations	50 (43%)	32 (41%)	20 (54%)	0.378
Compliant with CDC strength exercise recommendations	76 (66%)	31 (39%)	24 (65%)	0.001
Compliant with CDC strength and aerobic exercise recommendations	40 (34%)	14 (18%)	16 (43%)	0.007
Last visit with a primary care provider				0.368
Noncompliant >2 yr	33 (28%)	27 (34%)	8 (22%)	
Compliant <2 yr	83 (72%)	52 (66%)	29 (78%)	
Last visit with a dentist				0.067
Compliant <1 yr	12 (10%)	13 (16%)	1 (3%)	
Partially compliant 1-2 yr	82 (71%)	54 (68%)	32 (86%)	
Noncompliant >2 yrs	22 (19%)	12 (15%)	4 (11%)	
Last cholesterol check				0.142
<5 yr (compliant)	89 (77%)	60 (76%)	34 (92%)	
>5 yr (noncompliant)	22 (19%)	18 (23%)	1 (3%)	
Completed age-appropriate colon cancer screening				0.215
Not indicated	43 (37%)	35 (44%)	11 (30%)	
Indicated but not completed	10 (9%)	12 (15%)	4 (11%)	
Completed	63 (54%)	32 (41%)	22 (59%)	
Feelings of emotional exhaustion	20 (17%)	19 (24%)	10 (27%)	0.303
Feelings of depersonalization	21 (18%)	15 (19%)	11 (30%)	0.302
Burnout	14 (12%)	11 (14%)	8 (22%)	0.349

CDC = Centers for Disease Control and Prevention

**Table 4:** Health Habits, Routine Medical Care, and Burnout of Practicing Orthopaedic Surgeon According to the Number of Years in Practice^a^

Health and Wellness Practice	<5 yr (n = 41)	5-10 yr (n = 29)	10-20 yrs (n = 50)	>20 yr (n = 113)	*P* Value
Compliant with CDC aerobic exercise recommendations	21 (51%)	10 (34%)	20 (40%)	52 (46%)	0.487
Compliant with CDC strength exercise recommendations	25 (61%)	14 (48%)	29 (58%)	64 (57%)	0.758
Compliant with CDC strength and aerobic exercise recommendations	15 (37%)	7 (24%)	13 (26%)	36 (32%)	0.605
Last visit with a primary care provider					<0.001
Noncompliant >2 yr	20 (49%)	14 (48%)	19 (38%)	15 (13%)	
Compliant <2 yr	21 (51%)	15 (52%)	31 (62%)	98 (87%)	
Last visit with a dentist					0.201
Compliant <1 yr	24 (59%)	20 (69%)	38 (76%)	87 (77%)	
Partially compliant 1-2 yr	9 (22%)	3 (10%)	7 (14%)	17 (15%)	
Noncompliant >2 yr	8 (20%)	5 (17%)	5 (10%)	8 (7%)	
Last cholesterol check					<0.001
<5 yrs (compliant)	26 (63%)	18 (62%)	38 (76%)	102(90%)	
>5 yrs (noncompliant)	14 (35%)	9 (31%)	12 (24%)	6 (6%)	
Age-appropriate colon cancer screening					<0.001
Not indicated	39 (95%)	24 (83%)	24 (48%)	3 (3%)	
Indicated but not completed	0 (0%)	3 (10%)	10 (20%)	13 (12%)	
Completed	2 (5%)	2 (7%)	16 (32%)	97 (86%)	
Feelings of emotional exhaustion	7 (17%)	5 (17%)	13 (26%)	24 (21%)	0.709
Feelings of depersonalization	9 (22%)	9 (31%)	9 (18%)	20 (18%)	0.427
Burnout	5 (12%)	5 (17%)	6 (12%)	17 (15%)	0.887

CDC = Centers for Disease Control and Prevention

a Missing survey responses not included (<3%).

**Table 5:** Health Habits, Routine Medical Care, and Burnout of Practicing Orthopaedic Surgeon by Subspecialty

Health and Wellness Practice	Spine (n = 13)	Pediatrics (n = 11)	Trauma (n = 9)	Oncology (n = 4)	Sports Medicine (n = 33)	Hand (n = 41)	Shoulder + Elbow (n = 14)	Hip and Knee (n = 71)	Foot + Ankle (n = 14)	General (n = 21)	*P* Value
Compliant with CDC aerobic exercise	5 (38%)	5 (45%)	3 (33%)	0 (0%)	14 (42%)	23 (56%)	5 (36%)	31 (44%)	9 (64%)	9 (43%)	0.667
Compliant with CDC strength exercise	7 (54%)	10 (91%)	4 (44%)	1 (25%)	24 (73%)	20 (49%)	5 (36%)	40 (56%)	9 (65%)	13 (62%)	0.82
Compliant with CDC strength and aerobic exercise	4 (31%)	5 (45%)	2 (22%)	0 (0%)	13 (39%)	12 (29%)	2 (14%)	21 (30%)	6 (43%)	7 (33%)	0.747
Last visit with a primary care provider											0.586
Noncomplaint >2 yr	3 (23%)	5 (45%)	5 (56%)	1 (25%)	9 (27%)	11 (27%)	5 (36%)	21 (30%)	5 (36%)	3 (14%)	
Compliant <2 yr	10 (77%)	6 (55%)	4 (44%)	3 (75%)	24 (73%)	30 (73%)	9 (64%)	50 (70%)	12 (86%)	18 (86%)	
Last visit with a dentist											0.917
Compliant <1 yr	2 (15%)	0 (0%)	2 (22%)	0 (0%)	2 (6%)	5 (12%)	4 (29%)	9 (13%)	1 (7%)	1 (5%)	
Partially compliant 1-2 years	9 (69%)	9 (82%)	7 (78%)	4 (100%)	23 (70%)	28 (68%)	7 (50%)	52 (73%)	13 (93%)	17 (81%)	
Noncompliant >2 yr	2 (15%)	2 (18%)	0 (0%)	0 (0%)	7 (21%)	7 (17%)	3 (21%)	9 (13%)	3 (21%)	3 (14%)	
Last cholesterol check											0.759
<5 yr (compliant)	10 (77%)	9 (82%)	7 (78%)	3 (75%)	26 (79%)	31 (76%)	10 (71%)	54 (76%)	13 (93%)	21 (100%)	
>5 yr (noncompliant)	1 (8%)	2 (18%)	2 (22%)	1 (25%)	6 (18%)	7 (16%)	3 (21%)	15 (21%)	4 (29%)	0 (0%)	
Completed age-appropriate colon cancer screening											0.344
Not indicated	4 (31%)	4 (36%)	3 (33%)	4 (100%)	11 (33%)	13 (32%)	7 (50%)	34 (48%)	6 (43%)	4 (19%)	
Indicated but not completed	0 (0%)	2 (18%)	1 (11%)	0 (0%)	5 (15%)	6 (15%)	2 (14%)	7 (10%)	1 (7%)	2 (10%)	
Completed	9 (69%)	5 (45%)	5 (56%)	0 (0%)	17 (52%)	21 (51%)	5 (36%)	30 (42%)	10 (71%)	15 (71%)	
Feelings of emotional exhaustion	1 (8%)	3 (27%)	0 (0%)	1 (25%)	8 (24%)	8 (20%)	2 (14%)	19 (27%)	3 (21%)	4 (19%)	0.743
Feelings of depersonalization	3 (23%)	1 (9%)	3 (33%)	1 (25%)	5 (15%)	7 (17%)	1 (7%)	18 (25%)	4 (29%)	4 (19%)	0.79
Burnout	1 (8%)	1 (9%)	0 (0%)	1 (25%)	4 (12%)	6 (15%)	1 (7%)	13 (18%)	3 (21%)	3 (14%)	0.926

CDC = Centers for Disease Control and Prevention

### Routine Medical Care

Most orthopaedic surgeons indicated appropriate completion of primary care provider evaluation within 3 years (71%), dental visit within 2 years (84%), and cholesterol check within 5 years (79%). Similarly, most reported undergoing their indicated screenings for colorectal cancer (89%) and breast cancer (92% for age-appropriate female orthopaedic surgeons). No significant difference in compliance with any routine medical care or screening was noted for practice type (*P* > 0.2), number of years in practice (*P* > 0.2), or subspecialty (*P* > 0.3). However, the number of years in practice was inversely proportional to the degree of compliance with primary care provider evaluation: 87% of surgeons in practice for >20 years had a primary care visit versus 51% in those practicing <5 years (*P* < 0.001), and 90% of surgeons in practice for >20 years had a cholesterol check <5 years versus 63% in those practicing <5 years (*P* < 0.001).

### Wellness Strategies and Burnout

The most essential wellness strategies for orthopaedic surgeons included protecting time away from work to spend with family and friends (80%), finding meaning in work (70%), and regularly participating in recreation/exercise (64%). All three of these strategies were consistently ranked in this order of importance regardless of practice type, number of years in practice, or subspecialty. Both emotional exhaustion and depersonalization were reported at 21% and resulted in an overall burnout rate of 15% for orthopaedic surgeon survey responders. Burnout was most common in hospital practice type (22%), practices of 5 to 10 years (17%), and oncology subspecialty (25%); however, none of the subgroups demonstrated a significant association with burnout (*P* > 0.4).

## Discussion

Wellness is a multidimensional entity that includes physical, emotional, mental, and spiritual components, yet physical health may have an extraordinary effect on the risk of burnout. Improving one's physical health translates into beneficial effects on work-related fatigue, stress reduction, resilience, and self-efficacy—each of which is protective again burnout.^29,30^ This survey study benchmarked health habits, routine medical care, wellness strategies, and burnout in a cohort of actively practicing orthopaedic surgeons.

Despite stereotypes associated with the orthopaedic subspecialty, only 31% of surgeons surveyed met both CDC aerobic and strengthening exercise recommendations. Furthermore, surgeons in academic practice were at the most significant risk of not achieving enough exercise. Shanafelt et al^30^ reported similar findings whereby 55% of orthopaedic surgeons met CDC guidelines for physical activity. Unsurprisingly, those who met guidelines also demonstrated a higher quality of life and lower burnout. Another series reported a more optimistic rate of 62% of surgeons exercising three times per week and only 10% failing to complete any exercise.^31^ The reasons for limited physical fitness participation are personal, multifactorial, and cannot be elicited from basic survey results; however, we think that surgeons may feel burdened by the ever-increasing clinical and administrative demands regardless of practice type.

The survey cohort appeared to report excellent compliance with routine medical evaluations (71%) and preventive screening recommendations (79% to 89%). Harms et al^31^ reported similar results in a study of former general surgery residents spanning 25 years from a single academic program. The surgeons surveyed followed basic screening healthcare recommendations, including cardiac evaluation in 73%, prostate evaluation in 81%, and colonoscopy in 73%. Although no analysis of burnout was done, 75% of surgeons were satisfied with their health, practice, and career. In contrast, Shanafelt et al^30^ noted only 46% of over 7100 surgeons surveyed reported primary care evaluation in the last 12 months. These surgeons were also most likely to be compliant with age-appropriate healthcare screening tests and had superior healthcare quality of life scores. Nevertheless, the importance of establishing routine exercise habits and medical care cannot and should not be ignored by orthopaedic surgeons.

Beyond physical and medical health, psychological wellness is critical in preventing personal burnout. The universally highest-ranked wellness strategy from survey responders was recognized to be protecting personal time away from work to spend with family and friends. Increasing such time with family/friends, parenthood, and living with a significant other has been shown to decrease emotional exhaustion and improve one's sense of personal accomplishment.^3,12,13,32-34^ Conversely, poor work-life balance, family conflict, lack of spousal support, and a poor marital relationship have been found to contribute to emotional exhaustion and burnout.^5,30,34^ The second most important wellness strategy for orthopaedic surgeons was finding meaning in work. Satisfactory meaning in one's work must be protected by conflicts within workload, control, reward, community support, fairness, or personal values—all rooted in a perceived lack of autonomy.^1,35^ Other wellness strategies of participation in recreational activities and taking vacations were also highly ranked, which are integrally associated with lower rates of emotional exhaustion.^33,34,36^

The burnout rate was estimated to be 15% among orthopaedic surgeon survey responders, perhaps due to adherence to healthy habits and utilization of wellness strategies. This rate is considerably lower than the reported 32% to 57% rate of orthopaedic surgeon burnout from previous studies.^8,9,10,11,12,13^ The relatively low rate in our cohort may be a function of the adapted assessment tool, although the two-question MBI has previously been well validated against the full assessment.^26,28^ Our study also did not find significant differences between burnout and the subgroups of practice type, years in practice, or subspecialty. The current literature regarding the effect of these demographics is controversial and varied. In a nationwide survey of 64 orthopaedic programs, academic faculty reported high stress levels and difficulty with work-life balance; however, those with research duties were less likely to burnout.^36^ This stress may be secondary to a sense of accomplishment experienced, resulting in finding deeper meaning and passion in their career.^37^ Indeed, 34% of the survey cohort indicated academic practice type and could have lessened this cohort's burnout rate.

Contrary to academic faculty, orthopaedic surgery residents are especially prone to features of burnout.^33,36^ Specifically, emotional exhaustion and depersonalization peaked in postgraduate year 2 residents but did improve with increasing years of independent practice.^15,36^ In another large national cross-sectional survey of residents and faculty, posttraumatic stress disorder, poor work-life balance, and burnout were present but did eventually improve with increasing level of experience.^38^ Regardless of our survey findings, orthopaedic surgeon burnout remains a significant challenge, and surgeon wellness programs should be implemented early in residency to cultivate a healthy career and life.

There are multiple limitations to a self-reported survey study. Although greater than 700 surveys were sent electronically, the completion rate was only 30% despite three weeks of repetitive reminders for participation. Given the smaller numbers available for subgroup analysis, a limited statistical inference could be drawn. Moreover, the unequal distribution of demographic groups may predispose to selection bias (those who choose to participate in the survey may not be fully representative of the sample) and further limit the strength of conclusions. This response rate is not different from other electronic survey studies but indicates a future potential to obtain a more robust sample.^39^ Next, the responses were primarily benchmarked against CDC or USPTF recommendations, whereby other health agencies may provide different recommendations. Furthermore, the strength of preventive screening recommendations for colorectal or breast cancer is varied and subject to provider and/or patient interpretation of risks. The validated two-question adapted MBI was used to evaluate burnout in place of the 22-question inventory to mitigate survey fatigue; however, it is possible that this adapted assessment may fail to capture all aspects of burnout fully. In addition, the exercise and routine medical care results cannot be externally validated; rather, we rely on the responder's honest assessment of their habits. All survey answers are subject to recency bias—a cognitive bias that gives greater importance to the most recent events. Finally, the surveys were obtained during the ongoing COVID-19 pandemic, and the effect on personal and clinical practices could bias the results. Lazarides et al^40^ found no correlation of stressors related to the COVID-19 pandemic and orthopaedic physician burnout or fulfillment. Still, surgeon physical wellness and self-care remains an important topic with increasing priority as result of the pandemic.

In conclusion, most orthopaedic surgeons are not fully compliant with aerobic and strength exercise recommendations; however, they do generally participate in routine medical and health screen evaluation. According to our study, the most important self-identified wellness strategy is the protection of personal time, and the overall burnout rate was 15%. Orthopaedic surgeons should be encouraged to reflect on their health and wellness habits to enjoy career satisfaction and avoid burnout. This limited survey study highlights a critical topic of surgeon wellness, and future investigations and advocacy are strongly recommended.
